# Supplementary material for: Ecological indices of phytophagous Hemiptera and their natural enemies on Acacia auriculiformis (Fabales: Fabaceae) plants with or without dehydrated sewage sludge application in a degraded area
Source: PLoS One. 2020 Aug 17;15(8):e0237261. doi: 10.1371/journal.pone.0237261 (PMC7430702; doi:10.1371/journal.pone.0237261)
Supplement: S1 File — (DOCX) [file pone.0237261.s001.docx]

# Species of phytophagous Hemiptera, Sternorrhyncha predators, and protocooperating ants on *Acacia auriculiformis* plants

| Phytophagous Hemiptera | |
| --- | --- |
| Achilidae | Non-identified |
| Aleyrodidae | Non-identified |
| Aethalionidae | *Aethalion reticulatum* L. |
| Cicadellidae | *Acrogonia* sp. |
|  | *Balclutha hebe* Kirkaldy |
|  | *Erythrogoia sexguttata* F. |
| Cicadidae | *Quesada gigas* Oliver |
| Coreidae | *Leptoglossus* sp. |
| Fulgoridae | Non-identified |
| Membracidae | Non-identified |
|  | *Membracis* sp. |
| Nogodinidae | Non-identified |
| Pentatomidae | Non-identified |
| Scutelleridae | *Pachycoris torridus* Scopoli |
| Sternorrhyncha predators | |
| Coleoptera: Cantharidae | *Cantharis* sp. |
| Coccinellidae | *Cycloneda sanguinea* L. |
| Diptera: Syrphidae | *Syrphus* sp. |
| Dolichopodidae | Non-identified |
| Neuroptera: Chrysopidae | *Chrysoperla* sp. |
| Protocooperating ants | |
| Hymenoptera: Formicidae | *Brachmyrmex* sp. |
|  | *Camponotus* sp. |
|  | *Cephalotes* sp. |
|  | *Ectatomma* sp. |
|  | *Pheidole* sp. |
|  | *Pseudomyrmex termitarius* Smith |
