# Supplementary material for: Ecological indices of phytophagous Hemiptera and their natural enemies on Acacia auriculiformis (Fabales: Fabaceae) plants with or without dehydrated sewage sludge application in a degraded area
Source: PLoS One. 2020 Aug 17;15(8):e0237261. doi: 10.1371/journal.pone.0237261 (PMC7430702; doi:10.1371/journal.pone.0237261)
Supplement: Attachment — Submitted filename: PONE-D-20-08541_reviewer.pdf [file pone.0237261.s003.pdf]

// PLOS ONE

Can Acacia auriculiformis (Fabales: Fabaceae) fertilized with dehydrated sewage sludge contribute to the regeneration of degraded areas?

--Manuscript Draft--

**Manuscript Number:**	PONE-D-20-08541
**Article Type:**	Research Article
**Full Title:**	Can Acacia auriculiformis (Fabales: Fabaceae) fertilized with dehydrated sewage sludge contribute to the regeneration of degraded areas?
**Short Title:**	Can Acacia auriculiformis contribute to the regeneration of degraded areas?
**Corresponding Author:**	Germano Leão Demolin Leite, Ph.D.<br>Universidade Federal de Minas Gerais<br>Montes Claros, Minas Gerais BRAZIL
**Keywords:**	Biogeographic island, Hemiptera; Nitrogen; Protocooperating ants
**Abstract:**	Soil fertilization with dehydrated sewage sludge accelerates the recovery process of degraded areas through improved nutrient concentration, what favors the development of trophic webs with pioneer plants such as Acacia auriculiformis A. Cunn. ex Beth (Fabales: Fabaceae), phytophagous Hemiptera, predators, and protocooperanting ants. This study aimed to evaluate the development and production of A. auriculiformis litter fertilized with dehydrated sewage sludge and the ecological indices of sucking insects (Hemiptera), their predators and protocooperating ants, as bioindicators, in a degraded area for 24 months. Complete randomization was applied for two treatments (with or without dehydrated sewage sludge fertilization) in 24 replications (one repetition = one plant). Leaves/branch and branches/plant numbers, percentage of soil cover (litter), ecological indices of Phytophagous Hemiptera, their predators, and protocooperating ants were evaluated. The plants of A. auriculiformis fertilized with dehydrated sewage sludge revealed superior development when compared to the non-fertilized ones. The highest abundance and richness of phytophagous Hemiptera species and Sternorrhyncha predators occurred in fertilized A. auriculiformis plants. The increase in richness of species of protocooperanting ants that established mutualistic relationships positively influenced the phytophagous Hemiptera. The use of A. auriculiformis fertilized with dehydrated sewage sludge can recover of degraded areas due to its higher soil cover (e.g., litter) what, in general, results in higher ecological indices of phytophagous Hemiptera and their predators.
**Order of Authors:**	Luan Rocha Dourado
	Germano Leão Demolin Leite, Ph.D.
	Marcus Alvarenga Soares
	Gustavo Leal Teixeira
	Farley William Souza Silva
	Regynaldo Arruda Sampaio
**Additional Information:**	
**Question**	**Response**
**Financial Disclosure**<br><br>Enter a financial disclosure statement that describes the sources of funding for the work included in this submission. Review the submission guidelines for detailed requirements. View published research articles from *PLOS ONE* for specific examples.	NO - Include this sentence at the end of your statement: The funders had no role in study design, data collection and analysis, decision to publish, or preparation of the manuscript.

This statement is required for submission and **will appear in the published article** if the submission is accepted. Please make sure it is accurate.<br><br>**Unfunded studies**<br>Enter: *The author(s) received no specific funding for this work.*<br><br>**Funded studies**<br>Enter a statement with the following details:<br>• Initials of the authors who received each award<br>• Grant numbers awarded to each author<br>• The full name of each funder<br>• URL of each funder website<br>• Did the sponsors or funders play any role in the study design, data collection and analysis, decision to publish, or preparation of the manuscript?<br>• **NO** - Include this sentence at the end of your statement: *The funders had no role in study design, data collection and analysis, decision to publish, or preparation of the manuscript.*<br>• **YES** - Specify the role(s) played.<br><br>* typeset	
**Competing Interests**<br><br>Use the instructions below to enter a competing interest statement for this submission. On behalf of all authors, disclose any competing interests that could be perceived to bias this work—acknowledging all financial support and any other relevant financial or non-financial competing interests.<br><br>This statement **will appear in the published article** if the submission is accepted. Please make sure it is accurate. View published research articles from *PLOS ONE* for specific examples.	The authors have declared that no competing interests exist.

Powered by Editorial Manager® and ProduXion Manager® from Aries Systems Corporation

**NO authors have competing interests**<br><br>Enter: *The authors have declared that no competing interests exist.*<br><br>**Authors with competing interests**<br><br>Enter competing interest details beginning with this statement:<br><br>*I have read the journal's policy and the authors of this manuscript have the following competing interests: [insert competing interests here]*<br><br>* typeset	
**Ethics Statement**<br><br>Enter an ethics statement for this submission. This statement is required if the study involved:<br><br>• Human participants<br>• Human specimens or tissue<br>• Vertebrate animals or cephalopods<br>• Vertebrate embryos or tissues<br>• Field research<br><br>Write "N/A" if the submission does not require an ethics statement.<br><br>General guidance is provided below. Consult the submission guidelines for detailed instructions. **Make sure that all information entered here is included in the Methods section of the manuscript.**	N/A

Powered by Editorial Manager® and ProduXion Manager® from Aries Systems Corporation

**Format for specific study types**<br><br>**Human Subject Research (involving human participants and/or tissue)**<br>• Give the name of the institutional review board or ethics committee that approved the study<br>• Include the approval number and/or a statement indicating approval of this research<br>• Indicate the form of consent obtained (written/oral) or the reason that consent was not obtained (e.g. the data were analyzed anonymously)<br><br>**Animal Research (involving vertebrate animals, embryos or tissues)**<br>• Provide the name of the Institutional Animal Care and Use Committee (IACUC) or other relevant ethics board that reviewed the study protocol, and indicate whether they approved this research or granted a formal waiver of ethical approval<br>• Include an approval number if one was obtained<br>• If the study involved *non-human primates*, add *additional details* about animal welfare and steps taken to ameliorate suffering<br>• If anesthesia, euthanasia, or any kind of animal sacrifice is part of the study, include briefly which substances and/or methods were applied<br><br>**Field Research**<br><br>Include the following details if this study involves the collection of plant, animal, or other materials from a natural setting:<br>• Field permit number<br>• Name of the institution or relevant body that granted permission	
**Data Availability**<br><br>Authors are required to make all data underlying the findings described fully available, without restriction, and from the time of publication. PLOS allows rare exceptions to address legal and ethical concerns. See the PLOS Data Policy and FAQ for detailed information.	Yes - all data are fully available without restriction

A Data Availability Statement describing where the data can be found is required at submission. Your answers to this question constitute the Data Availability Statement and **will be published in the article**, if accepted.<br><br>**Important:** Stating ‘data available on request from the author’ is not sufficient. If your data are only available upon request, select ‘No’ for the first question and explain your exceptional situation in the text box.<br><br>Do the authors confirm that all data underlying the findings described in their manuscript are fully available without restriction?	
**Describe where the data may be found in full sentences. If you are copying our sample text, replace any instances of XXX with the appropriate details.**<br><br>• If the data are **held or will be held in a public repository**, include URLs, accession numbers or DOIs. If this information will only be available after acceptance, indicate this by ticking the box below. For example: *All XXX files are available from the XXX database (accession number(s) XXX, XXX.)*.<br>• If the data are all contained **within the manuscript and/or Supporting Information files**, enter the following: *All relevant data are within the manuscript and its Supporting Information files.*<br>• If neither of these applies but you are able to provide **details of access elsewhere**, with or without limitations, please do so. For example:<br><br>*Data cannot be shared publicly because of [XXX]. Data are available from the XXX Institutional Data Access / Ethics Committee (contact via XXX) for researchers who meet the criteria for access to confidential data.*<br><br>*The data underlying the results presented in the study are available from (include the name of the third party*	All relevant data are within the manuscript and its Supporting Information files.

and contact information or URL).<br>• This text is appropriate if the data are owned by a third party and authors do not have permission to share the data.<br><br>* typeset	
Additional data availability information:	Tick here if the URLs/accession numbers/DOIs will be available only after acceptance of the manuscript for publication so that we can ensure their inclusion before publication.

Powered by Editorial Manager® and ProduXion Manager® from Aries Systems Corporation

Manuscript

Click here to access/download;Manuscript;A. auriculiformis Hemiptera.pdf

Can *Acacia auriculiformis* (Fabales: Fabaceae) fertilized with dehydrated sewage sludge contribute to the regeneration of degraded areas?

Luan R. Dourado[1], Germano L.D. Leite[2]*, Marcus A. Soares[1], Gustavo L. Teixeira[2], Farley W.S. Silva[3], Regynaldo A. Sampaio[2]

[1]Departamento de Agronomia, Universidade Federal dos Vales do Jequitinhonha e Mucuri, Diamantina, Minas Gerais, Brasil

[2]Instituto de Ciências Agrárias, Universidade Federal de Minas Gerais, Montes Claros, Minas Gerais, Brasil

[3]Departamento de Agronomia, Universidade Federal do Acre, Rio Branco, Acre, Brasil

*Corresponding author

E-mail: germano.demolin@gmail.com (GLDL)

ABSTRACT

Soil fertilization with dehydrated sewage sludge accelerates the recovery process of degraded areas through improved nutrient concentration, what favors the development of trophic webs with pioneer plants such as *Acacia auriculiformis* A. Cunn. ex Beth (Fabales: Fabaceae), phytophagous Hemiptera, predators, and protocooperanting ants. This study aimed to evaluate the development and production of *A. auriculiformis* litter fertilized with dehydrated sewage sludge and the ecological indices of sucking insects (Hemiptera), their predators and protocooperating ants, as bioindicators, in a degraded area for 24 months. Complete randomization was applied for two treatments (with or without dehydrated sewage sludge fertilization) in 24 replications (one repetition = one plant). Leaves/branch and branches/plant numbers, percentage of soil cover (litter), ecological indices of Phytophagous Hemiptera, their predators, and protocooperating ants were evaluated. The plants of *A. auriculiformis* fertilized with dehydrated sewage sludge revealed superior development when compared to the non-fertilized ones. The highest abundance and richness of phytophagous Hemiptera species and Sternorrhyncha predators occurred in fertilized *A. auriculiformis* plants. The increase in richness of species of protocooperanting ants that established mutualistic relationships positively influenced the phytophagous Hemiptera. The use of *A. auriculiformis* fertilized with dehydrated sewage sludge can recover of degraded areas due to its higher soil cover (e.g., litter) what, in general, results in higher ecological indices of phytophagous Hemiptera and their predators.

Keywords: Biogeographic island, Hemiptera; Nitrogen; Protocooperating ants.

Introduction

Tropical areas soils (eg., Brazil), in general, are highly weathered, present poor chemical quality and fragile macrostructure [1]. Moreover, the tropical climate, with high temperatures and humidity, accelerates the degradation of soil organic matter [2]. These factors, combined with poor soil management, forest clearing and burning, intensive mechanization, and grazing, promote changes in ecosystems at a faster rate than the natural regeneration capacity [2-4]. A destabilized ecosystem, in turn, negatively affects species richness, abundance, and distribution [5, 6]. Therefore, depending on the intensity of soil degradation, the use of restoration techniques is suggested for fauna and flora rehabilitation [7].

In this sense, species of the genus *Acacia* (Fabales: Fabaceae) may be useful due to their rapid growth and the capacity for biological nitrogen fixation (BNF) by association with symbiotic bacteria [8]. Also, the natural introduction of nitrogen (N) can intensify the cycle of other nutrients and stabilize soil organic matter in degraded environments [9]. Among the species of this genus, *Acacia auriculiformis* A. Cunn. ex Beth stands out for its resilience, lower susceptibility to disease, and adaptability [10-12]. In addition to BNF, *A. auriculiformis* provides other ecosystem services such as moisture retention, potassium deposition, soil organic carbon (litter) and heavy metal phytoextraction with mycorrhizal associations [13-18].

Among the varieties of waste produced by anthropological activity, sewage sludge production stands out as a by-product of urban wastewater treatment facilities [19]. In Brazil, dehydrated sewage sludge is used in agriculture (e.g., *Saccharum* sp. L. (Poaceae) and *Phaseolus vulgaris* L. (Faboideae)) in reforestation (e.g., *Acacia mangium* Willd. (Fabaceae) and *Pittosporum tenuifolium* Sol. Ex Gaertn. (Pittosporaceae)) as a fertilizer and soil conditioning agent [20-26]. As it holds significant amounts of organic matter and nutrients (e.g., nitrogen and phosphorus), sewage sludge improves plant development and the physical, chemical, and biological properties of the soil [27, 28]. However, due to the high concentration of nutrients, the presence of pathogens, heavy metals and persistent organic pollutants, the inappropriate disposal of sewage sludge can cause environmental impacts [29, 30]. Also, sewage sludge, through its nutrients, can impact insect population where N levels are above or below ideal, affecting the physiology, diversity, and distribution of phytophagous insects [26, 31-33]. The ecological indices of these species can be employed to monitor the recovery of degraded areas due to its great diversity, amount of occupied habitats, importance in biological processes and rapid response to environmental changes [34, 35]. Insects of the orders Hemiptera (e.g., Cicadidae) and Hymenoptera (e.g., Formicidae), for instance, are used as bioindicators of

degraded areas recovery [36, 37]. In this context, plants that grow vigorously are more susceptible to attacks of herbivorous insects - Plant Vigor Hypothesis - generating greater diversity and abundance of insects and therefore, natural enemies [38]. Under these conditions, the same ecological processes of the theory of biogeographic islands (BGI) apply to plants, with a higher probability of extinction of rarer species in smaller BGI [39-41].

Thus, the objective of this study was to evaluate the development and soil cover by *A. auriculiformis* fertilized with sewage sludge and ecological indices of phytophagous Hemiptera, Sternorrhyncha predators and protocooperating ants, as bioindicators, in a degraded area by testing two hypotheses: i) fertilized plants will have larger crowns and form more litter, thus assisting in the recovery of degraded soils [26, 42, 43] and ii) fertilized plants will be larger (> BGI) and with better nutritional quality (> free amino acids), greater abundance, species richness and diversity of phytophagous Hemiptera and, consequently, predators of Sternorrhyncha and protocooperating ants [41, 44, 45].

Results

Effect of fertilization on Acacia auriculiformis plants

The plants of *A. auriculiformis* fertilized with dehydrated sewage sludge presented higher numbers of leaves/branch, branches/plant and percentage of soil cover (eg., litter) ($P < 0.05$) (Table 1).

Insect Ecological Indexes

The highest abundance, diversity, and species richness ($P < 0.05$) of phytophagous Hemiptera and Sternorrhyncha predators occurred in fertilized *A. auriculiformis* plants. However, the ecological indices of protocooperating ants did not differ statistically ($P > 0.05$) between the treatments. The increase of leaves/branch and branches/plant affected positively the species richness of phytophagous Hemiptera, abundance, species richness and species diversity of Sternorrhyncha predators, and the abundance of protocooperating ants. Enhancement in abundance and richness of protocooperating ants species positively influenced the same parameters of phytophagous Hemiptera, and vice versa (Tables 1-2).

Discussion

Acacia auriculiformis plants fertilized with dehydrated sewage sludge presented higher crowns, with an increase in leaves (> 6.7) and branches (> 12.2) production per plant, providing a more significant litter deposition, which provided reduction of kinetic energy from rainfall and soil temperature oscillation, also improving nutrient cycling and moisture retention[46, 47]. Such fact confirms the first hypothesis that fertilized plants will be larger and with higher litter

deposition, what enhances the recovery process of degraded areas [48]. Since it contains organic matter, macro, and micronutrient contents, dehydrated sewage sludge provides better conditions for the growth and development of *A. auriculiformis* [28, 49, 50]. So much as with *A. mangium* Willd. (Fabaceae) [26], *Cordyline australis* (G. Forst.) Endl (Asparagaceae), *Eucalyptus grandis* Hill (Myrtaceae), *Lafoensia pacari* Saint-Hilaire (Lythraceae) and *Pittosporum tenuifolium* Sol. Eg. Gaertn. (Pittosporaceae) [22, 24, 42, 43]. Therefore, even though the recovery of degraded areas is a slow process [51], the usage of sewage sludge as fertilizer can accelerate the process. Besides, *A. auriculiformis* is promising in the recovery of degraded areas due to its pioneering nature, which guarantees assistance for the development of other species [52-54].

The plants of *A. auriculiformis* fertilized with dehydrated sewage sludge presented higher ecological indices of phytophagous Hemiptera and, consequently increased those of Sternorrhyncha predators as well as > leaves => protocooperating ants. Enhancement occurs due to larger canopy sizes when compared to non-fertilized plants, which confirms the second hypothesis:> BGI => ecological indices of phytophagous Hemiptera, and, consequently, of predators and protocooperating plants. Each plant alone behaves as a small-scale BGI, where those that grow rapidly and reach larger than average size are preferred by herbivores and are also subject to more complex plant-arthropod interactions (i.e., plant vigor hypothesis). [38-41, 55, 56]. Additional studies can prove the positive correlation between phytophagous and the boost of plant crown (> biomass> resources), for example, the abundance of phytophagous insects in *A. mangium* Wild. (Fabales: Fabaceae); galling insects in *Macairea radula* (Bonpl.) DC. (Myrtales: Melastomataceae); and *Carpatolechia proximella* Hbn. (Lepidoptera: Gelechiidae) in *Picea abies* (L.) Karst. (Pinaceae: Pinales) [26, 57-59]. On the other hand, the increase in nutrient availability, especially nitrogen, provided by fertilization, reflects upon the quality of sap (> amount of protein and free amino acids) [31, 45, 59, 60]. This increase benefits sucking insects that get enough nutrients to survive through the host plant sap (e.g., Sternorrhyncha), improving their performance and population density [61, 62]. Also, larger plants offer clearances from enemies due to greater size and architectural complexity, reflecting on the distribution of herbivorous insects [63, 64, 65]. The increase in habitat complexity by larger plants also provides indirect benefits to natural enemies, as it supports greater abundance of phytophages and increases the chances of rare species maintenance [39-41, 59, 66]. Thus, higher BGI affects a more significant number of predators in response to their prey abundance and lifestyle [40, 55, 67, 68]. Also, predatory insects generally have smaller population sizes than their prey; therefore, they must face a higher probability of local extinction, particularly in

smaller plants (<BGI) [69]. Some ant species establish symbiosis with numerous Sternorrhyncha species [70]. In places where ants feed on honeydew - sugary substances defecated by carbohydrate-rich aphids offer protection against natural enemies, also frightening other competing phytophages [71-73]. Thus, the presence of protocooperating ants reduces predators, competitors and encourages the presence of other species of Sternorrhyncha [74, 75].

Finally, as presented here, *A. auriculiformis* plants fertilized with dehydrated sewage sludge present higher crowns, resulting in increased litter deposition and ecological indices of phytophagous Hemiptera, predators, and protocooperating ants. Therefore, the recovery programs of degraded areas on this basis is promising.

Material and methods

Experimental site

The study was carried out in a degraded area at the "Instituto de Ciências Agrárias (ICA)" of the "Universidade Federal de Minas Gerais (UFMG)," Montes Claros city, Minas Gerais State, Brazil (latitude 16º51' S × longitude 44º55' W, altitude 943 m) from March 2017 to February 2019 (24 months; arthropod collection period). The area was defined as degraded due to soil losses and changes in soil chemistry or hydrology [76, 77]. The climate of this area is considered Aw: tropical savannah, with dry winters and rainy summers, according to the Köppen classification by [78], with annual precipitation between 1000 - 1300 mm and a yearly average temperature of $\geq$ 28ºC. The soil type is litolic neosoil [79] and chemical and physical details were described [26].

Experimental design

Acacia auriculiformis seeds were obtained from 5-year-old trees grown at ICA/UFMG. *Acacia auriculiformis* seedlings were grown in a nursery in plastic bags (8 x 12 cm) with a substrate mixture of 30% organic compost, 30% clay soil, 30% sand, and 10 % reactive natural phosphate (160 g)/molt in March 2016. The organic compost consists of three parts by volume: two parts of chopped prunings ($\leq$ 5 cm) and one part of tanned manure. The soil pH in the pits was rectified with dolomitic limestone (relative total neutralization power of 90%) (187 g/pit), increasing the base saturation to 50% [80]. Natural phosphate (80g/pit), fritted trace elements (FTE) (10g/pit), and marble dust (1kg/pit) were added according to the soil analysis. The *A. auriculiformis* seedlings were transplanted 30 cm high in pits (40 × 40 × 40 cm) two meters apart, in six parallel lines on flat ground (same characteristics). 24 plants were fertilized with 20 L of dehydrated sewage sludge/pit and 24 plants were not fertilized, in September 2016. The seedlings were irrigated twice a week until the beginning of the rainy season when no more

water was provided. The plants were pruned with a sterilized razor (each plant) when their branches reached 5 cm in length, cutting the additional stems and branches up to 1/3 of crown height, leaving out only the best stem. All pruned parts of the plants were left between planting lines. The design was completely randomized with two treatments (with dehydrated sewage sludge and without sewage sludge) with 24 replications (one repetition = one plant). The 20 L of dehydrated sewage sludge/pit was placed in a single dose.

Dehydrated sewage sludge (5% moisture) was collected at the “Estação de Tratamento de Esgoto (ETE)” in the city of Juramento, Minas Gerais, Brazil, about 40 km from the *A. auriculiformis* planting site. The ETE is operated by the “Companhia de Saneamento de Minas Gerais (COPASA)”. It can treat 217 m^3 of sewage sludge/day. The efficiency of the system in terms of organic matter removal is over 90%. The sewage sludge goes through a solarization process in coarse sand tanks for three months in the ETE reducing the thermotolerant coliform bacteria to levels accepted by the National Council for the Environment – “Conselho Nacional do Meio Ambiente (CONAMA)” (Resolution Nº 375) of the Ministry of the Environment – “Ministério do Meio Ambiente” of Brazil (http://www.mma.gov.br/port/conama/res/res06/res37506.pdf) for agricultural usage, which is $<10^3$ most likely number.g^{-1} of total solids. The main chemical and biological characteristics of the dehydrated sewage sludge of this company were described [49].

Plant mass production and soil coverage

Leaves/branch, branches/plant numbers, the percentage of soil cover by litter, grass and herbaceous plants were evaluated visually per month and plot (1.0 m^2) in the crown projection area of the 48 *A. auriculiformis* plants.

Insects

Insects were counted visually, fortnightly, between 7 and 11 am, on the adaxial and abaxial sides of leaves in the apical, middle and basal parts of the canopy and in the north, south, east and west directions, totaling 12 leaves/plant/evaluation, in each of the 48 six-month-old *A. auriculiformis* trees for 24 months. Insects were not removed from plants during evaluations. The total sampling effort was of 27648 leaves covering the entire plant (vertical and horizontal axes) for observation of as many insect species as possible, especially the rarest ones. At least three specimens per species of insects were captured by a vacuum cleaner, stored in 70% ethanol glass vials or assembled, broken down into morphospecies, and sent for identification (Supplementary material I).

Ecological indices

Ecological indices (species abundance, diversity, and species richness) were calculated for the species identified in the treatments (with or without dehydrated sewage sludge)/tree with BioDiversity Professional, Version 2 (© 1997 The Natural History Museum: http: //www.sams.ac.uk/dml/projects/benthic/bdpro/index.htm) [81]. Diversity was calculated with Hill's formula [82, 83] and species richness with the Simpson Index [84, 85].

Statistics

Leaves/branch, branches/plant data and percentage of soil cover per litter, herbaceous and grassy plants, abundance, diversity and species richness of Phytophagous Hemiptera, Sternorrhyncha predators and protocooperating ants were subjected to non-parametric statistical test, Wilcoxon signed-rank test ($P < 0.05$) [86] by the System for Statistical and Genetic Analysis - SAEG, version 9.1 [87]. Data were also submitted to simple regression or principal component regression (PCR) when linear ($P < 0.05$) to test the interactions between these groups of insects and *A. auriculiformis* total number of leaves and branches. The regression model known as PCR applies principal component analysis, based on a covariance matrix, to perform regression. Thus, it is possible to reduce the dimension of regression by the exclusion of the aspects that contribute to collinearity problems, or, linear relationships between the independent variables. All results were significant at ($P < 0.05$) for variable selection based on the stepwise method.

Ethics. No specific permits are required to plant *Acacia auriculiformis* in Brazil. The laboratory and field studies did not involve endangered or protected species.

Data accessibility. All data generated or analyzed during this study are included in this manuscript. Author’s contributions. The study was conceived and designed by L.R.D, G.L.D.L. and R.A.S., data were collected by L.R.D and F.W.S.S. with support from G.L.D.L. and R.A.S., data analysis was done by G.L.D.L. and M.A.S., figures, tables and manuscript preparation was done by L.R.D, G.L.D.L., M.A.S. and G.L.T. and all authors contributed to revisions and approve the final manuscript.

Competing interests. The authors declare that they have no conflict of interest.

Funding. The study was financially supported by the following Brazilian agencies “Conselho Nacional de Desenvolvimento Científico e Tecnológico (CNPq)”, “Coordenação de Aperfeiçoamento de Pessoal de Nível Superior (CAPES-Finance Code 001)”, “Fundação de Amparo à Pesquisa do Estado de Minas Gerais (FAPEMIG)”, and “Programa Cooperativo sobre Proteção Florestal (PROTEF)” of the “Instituto de Pesquisas e Estudos Florestais (IPEF)”.

Acknowledgements. We would like to thank to Dr. Carlos Matrangolo (UNIMONTES) (Formicidae), Dr. Ivan Cardoso Nascimento (EMBRAPA-ILHÉUS Cocoa Research Center, CEPLAC, Itabuna, BA) (Formicidae), Dr. Luci Boa Nova Coelho (Federal University of Rio de Janeiro (Cicadellidae) and Dr. Paulo Sérgio Fiuza Ferreira (Hemiptera) (UFV) for specimen identifications. Voucher numbers are 1595/02 and 1597/02 (CDZOO, Federal University of Paraná, Paraná, Brazil).

References

1. Gomes DS, Marciano CR, Faustino LL. Physical quality of a typichapludult soil under forest leguminous trees and pasture. Floresta e Ambiente. 2018; 25(4):1-8.

2. Osei-Tutu G, Abunyewa AA, Dawoe EK, Agbenyega O, Barnes RV. Effect of multipurpose trees and shrubs on degraded mined out soil in a semi-deciduous forest zone of West Africa. Land Degradation & Development. 2018; 29(1):3432–3439.

3. Nunes DAD, Gama-Rodrigues EF, Barreto PAB, Gama-Rodrigues AC, Monroe PHM. Carbon and nitrogen mineralization in soil of leguminous trees in a degraded pasture in northern Rio de Janeiro, Brazil. Journal of Forest Research. 2016; 27(1):91-99.

4. Rojas JM, Prause J, Sanzano GA, Arce OEA, Sánchez MC. Soil quality indicators selection by mixed models and multivariate techniques in deforested areas for agricultural use in NW of Chaco, Argentina. Soil & Tillage Research. 2016; 155(1):250-262.

5. Tibcherani M, Nacagava VAF, Aranda R, Mello RL. Review of Ants (Hymenoptera: Formicidae) as bioindicators in the Brazilian Savanna. Sociobiology. 2018; 65(2):112-129.

6. Pang D, Cao J, Dan X, Guan Y, Peng X, Cui M, et al. Recovery approach affects soil quality in fragile karst ecosystems of southwest China: Implications for vegetation restoration. Ecological Engineering. 2018; 123(1):151–160.

7. Macedo MO, Resende AS, Garcia PC, Boddey RM, Jantalia CP, Urquiaga S, et al. Changes in soil C and N stocks and nutrient dynamics 13 years after recovery of degraded land using leguminous nitrogen-fixing trees. Forest Ecology and Management. 2008; 255(1):1516–1524.

8. Hung TT, Doyle R, Eyles A, Mohammed C. Comparison of soil properties under tropical Acacia hybrid plantation and shifting cultivation land use in northern Vietnam. Southern Forests. 2016; 79(1):1-10.

9. Balieiro FC, Costa CA, Oliveira RB, Donagemma GK, Andrade AG, Capeche CL. Carbon stocks in mined area reclaimed by leguminous trees and sludge. Revista Árvore. 2017; 41(6):1-10.

10. Diouf D, Duponnoins R, Ba AT, Neyra M, Lesueur D. Symbiosis of *Acacia auriculiformis* and *Acacia mangium* with mycorrhizal fungi and *Bradyrhizobium* spp. improves salt tolerance in greenhouse conditions. Functional Plant Biology. 2005; 32(12):1143-1152.

11. Wong MML, Cannon CH, Wickneswail R. Identification of lignin genes and regulatory sequences involved in secondary cell wall formation in *Acacia auriculiformis* and *Acacia mangium* via de novo transcriptome sequencing. BMC Genomics. 2011; 342(12):1-13.

12. Rahman M, Rahman A, Miah G, Saha SR, Karim MA, Mostofa MG. Mechanistic insight into salt tolerance of *Acacia auriculiformis*: the importance of ion selectivity, osmoprotection, Tissue Tolerance, and Na^+ Exclusion. Frontiers in Plant Science. 2017; 8(1):1-15.

13. Huong VD, Nambiar EKS, Quanq LT, Mendham DS, Dung PT. Improving productivity and sustainability of successive rotations of *Acacia auriculiformis* plantations in South Vietnam. Southern Forests. 2015; 77(1):51–58.

14. Zheng M, Chen H, Li D, Zhu X, Zhang W, Fu S, et al. Biological nitrogen fixation and its response to nitrogen input in two mature tropical plantations with and without legume trees. Biology and Fertility of Soils. 2016; 52(1):665-674.

15. Zhu X, Chen H, Zhang W, Huang J, Fu S, Liu Z, et al. Effects of nitrogen addition on litter decomposition and nutrient release in two tropical plantations with N_2 fixing vs. non-N_2-fixing tree species. Plant Soil. 2016; 399(1):61-74.

16. Chen H, Chen M, Li D, Mao Q, Zhang W, Mo J. Responses of soil phosphorus availability to nitrogen addition in a legume and a non-legume plantation. Geoderma. 2018; 322(1):12-18.

17. Muthukumar T, Udaiyan K. Coinoculation of bioinoculants improve *Acacia auriculiformis* seedling growth and quality in a tropical Alfisol soil. Journal of Forestry Research. 2018; 29(3):663-673.

18. Rana V, Maiti SK. Differential distribution of metals in tree tissues growing on reclaimed coal mine overburden dumps, Jharia coal field (India). Environmental Science and Pollution Research. 2018; 25(1):9745–9758.

19. Zhou Y, He Y, He Y, Liu X, Xu B, Yu J, et al. Analyses of tetracycline adsorption on alkali-acid modified magnetic biochar: Site energy distribution consideration. Science of the Total Environment. 2018; 650(1):2260-2266.

20. Nascimento CWA, Barros DAS, Melo EEC, Oliveira AB. Alterações químicas em solos e crescimento de milho e feijoeiro após aplicação de lodo de esgoto. Revista Brasileira de Ciências do Solo. 2004; 28(2):385-392.

21. CONAMA – Conselho Nacional do Meio Ambiente. Resolução nº 375, de 29 de agosto de 2006. Brasília, 2006. 32p.

22. Caldeira Júnior CF, De Souza RA, Santos AM, Sampaio RA, Martins ER. Características químicas do solo e crescimento de *Astronium fraxinifolium* Schott em área degradada adubada com lodo de esgoto e silicato de cálcio. Ceres. 2009; 56(2):213-218.

23. Franco A, Abreu Junior CH, Perecin D, Oliveira FC, Granja ACR, Braga VS. Sewage sludge as nitrogen and phosphorus source for cane-plant and first ratoon crops. Revista Brasileira de Ciências do Solo. 2010; 34(2):553-561.

24. Gutiérrez-Ginés MJ, Robinson BH, Esperschuetz J, Madejón E, Horswell J, Mclenaghen R. Potential Use of Biosolids to Reforest Degraded Areas with New Zealand Native Vegetation. Journalof Environmental Quality. 2017; 46(4):906-914.

25. Limam RD, Limam I, Clerandeauc C, Khouatmia M, Djebali W, Cachot J, et al. Assessment of the toxicity and the fertilizing power from application of gamma irradiated anaerobic sludge as fertilizer: Effect on *Vicia faba* growth. Radiation Physics and Chemistry. 2018; 150(1):163-168.

26. Silva JL, Leite GLD, Tavares WS, Silva FWS, Sampaio RA, Azevedo AM, et al. Diversity of arthropods on *Acacia mangium* (Fabaceae) and production of this plant with dehydrated sewage sludge in degraded area. Royal Society Open Science. 2020; 7:1-12.

27. Aishah RM, Shamshuddin J, Fauziah CI, Arifin A, Panhwar QA. Phytoremediation of copper and zinc in sewage sludge amended soils using *Jatropha curcas* and *Hibiscus cannabinus*. Journal of the Chemical Society of Pakistan. 2016; 38(6):1230-1243.

28. Wu D, Feng J, Chu S, Jacobs DF, Tong X. Integrated application of sewage sludge, earthworms and *Jatropha curcas* on abandoned rare-earth mine land soil. Chemosphere. 2019; 214(1):47-54.

29. Martins MNC, Souza VV, Souza TS. Genotoxic and mutagenic effects of sewage sludge on higher plants. Ecotoxicology and Environmental Safety. 2016; 124(1):489-496.

30. Caldeira MVW, Favalessa M, Delarmelina WM, Gonçalves R dos SM. Sewage sludge assessment on growth of *Acacia mangium* seedlings by principal components analysis and orthogonal contrasts. Journal of Plant Nutrition. 2018; 41(10):1303-1311.

31. Leite GLD, Picanço M, Zanuncio JC, Moreira MD, Jham GN. Hosting capacity of horticultural plants for insect pests in Brazil. Chilean Journal of Agricultural Research. 2011; 71(3):383-389.

32. Katrínardóttir B, Alves JA, Sigurjónsdóttir H, Hersteinsson P, Gunnarsson TG. The effects of habitat type and volcanic eruptions on the breeding demography of icelandic whimbrels *Numenius phaeopus*. PloS One. 2015; 10(7):1-15.

33. Bownes A. Effects of *Hydrilla verticillata* (L.f.) Royle (Hydrocharitaceae) growing conditions and nutrient content on the performance of a leaf-mining fly, *Hydrellia purcelli* Deeming (Diptera: Ephydridae). Biocontrol Science and Technology. 2018; 28(3):1–15.

34. Wink C, Guedes JVC, Fagundes CK, Rovedder AP. Insetos edáficos como indicadores da qualidade ambiental. Revista de Ciências Agroveterinárias. 2005; 4(1):60-71.

35. Barah P, Bones AM. Multidimensional approaches for studying plant defence against insects: from ecology to omics and synthetic biology. Journal of Experimental Botany. 2015; 66(2):479-493.

36. Komonen A, Övermark E, Hytörnen J, Halme P. Tree species influences diversity of ground-dwelling insects in afforested fields. Forest Ecology and Management. 2015; 349(1):12-19.

37. Hou Z, Liu Y, Wei C. Influence of construction and demolition waste on fitness and community structure of cicada nymphs: New bioindicators of soil pollution. PloS One. 2018; 13(9):1-12.

38. Price PW. The plant vigor hypothesis and herbivore attack. Oikos. 1991; 62(2):244-251.

39. Kitahara M, Fujii K. An Island Biogeographical Approach to the Analysis of Butterfly Community Patterns in Newly Designed Parks. Researches on Population Ecology. 1997; 31(1):23-35.

40. Burns KC. Native–exotic richness relationships: a biogeographic approach using turnover in island plant populations. Ecological Society of America. 2016; 97(11):2932–2938.

41. Leite GLD, Veloso RV dos S, Zanuncio JC, Azevedo AM, Silva JL, Wilcken CF, et al. Architectural diversity and galling insects on *Caryocar brasiliense* trees. Scientific Reports. 2017; 7(1):1-7.

42. Silva PHM, Poggiani F, Laclau JP. Applying sewage sludge to *Eucalyptus grandis* plantations: effects on biomass production and nutrient cycling through litterfall. Applied and Environmental Soil Science. 2011; 2011(1):1-11.

43. Abreu AHM, Marzola LB, Melo LA, Leles PS dos S, Abel ELS, Alonso JM. Urban solid waste in the production of *Lafoensia pacari* seedlings. Revista Brasileira de Engenharia Agrícola e Ambiental. 2017; 21(2):83-87.

44. Schmitz OJ. Herbivory from Individuals to Ecosystems. Annual Review of Ecology, Evolution, and Systematics. 2008; 39(1):133-152.

45. Taiz L, Zeiger E, Moller IM, Murphy A. Fisiologia e desenvolvimento vegetal. 6. ed. Artmed. 2017.

46. Balieiro F de C, Dias LE, Franco AA, Campello EFC, Faria SM de. Acúmulo de nutrientes na parte aérea, na serapilheira acumulada sobre o solo e decomposição de filódios de *Acacia mangium* Willd. Ciência Florestal. 2004; 14(1):59-65.

47. Roundy BA, Farmer M, Olson J, Petersen S, Nelson DR, Davis J, et al. Runoff and sediment response to tree control and seeding on a high soil erosion potential site in Utah: evidence for reversal of an abiotic threshold. Ecohydrology. 2017; 10(1):1-9.

48. Bonini CSB, Alves MC, Montanari R. Lodo de esgoto e adubação mineral na recuperação de atributos químicos de solo degradado. Revista Brasileira de Engenharia Agrícola e Ambiental. 2015; 19(4):388–393.

49. Nogueira TAR, Sampaio RA, Fonseca IM, Ferreira CS, Santos SE, Ferreira LC, et al. Metais pesados e patógenos em milho e feijão caupi consorciados, adubados com lodo de esgoto. Revista Brasileira de Engenharia Agrícola e Ambiental. 2007; 11(3):331–338.

50. Hermassi M, Dosta J, Valderrama C, Lincon E, Morenoe N, Querol X, et al. Simultaneous ammonium and phosphate recovery and stabilization from urban sewage sludge anaerobic digestates using reactive sorbents. Science of the Total Environment. 2018; 630(1):781–789.

51. Modesto PT, Scabora MH, Colodro G, Maltoni KL, Cassiolato AMR. Alterações em algumas propriedades de um latossolo degradado com uso de lodo de esgoto e resíduos orgânicos. Revista Brasileira de Ciências do Solo. 2009; 33(5):1489-1498.

52. Azad S, Manik MR, Hasan S, Matin A. Effect of different pre-sowing treatments on seed germination percentage and growth performance of *Acacia auriculiformis*. Journal of Forestry Research. 2011; 22(2):183–188.

53. Peng SL, Liu J, Lu HF. Characteristics and role of *Acacia auriculiformis* on vegetation restoration in lower subtropics of china. Journal of Tropical Forest Science. 2005; 17(4):508-525.

54. Tham MW, Liew KC. Influence of different extraction temperatures and methanol solvent percentages on the total phenols and total flavonoids from the heartwood and bark of *Acacia auriculiformis*. European Journal of Wood and Wood Products. 2014; 72(1):67-72.

55. Ferrier SM, Price PW. Oviposition preference and larval performance of a rare bud galling sawfly (Hymenoptera: Tenthredinidae) on willow in northern Arizona. Environmental Entomolology. 2004; 33(1):700–708.

56. Heisswolf A, Obermaier E, Poethke HJ. Selection of large host plants for oviposition by a monophagous leaf beetle: nutritional quality or enemy-free space? Ecological Entomology. 2005; 30(1):299-306.

57. Lara DP, Oliveira LA, Azevedo IFP, Xavier MF, Silveira FAO, Carneiro MAA, et al. Relationships between host plant architecture and gall abundance and survival. Revista Brasileira de Entomologia. 2008; 52(1):78-81.

58. Zvereva E, Zverev V, Kozlov M. High densities of leaf-tiers in open habitats are explained by host plant architecture. Ecological Entomology. 2014; 39(1):470–479.

59. Silva FWS, Leite GLD, Guañabens REM, Sampaio RA, Gusmão CAG, Serrão JE, et al. Seasonal abundance and diversity of arthropods on *Acacia mangium* (Fabales: Fabaceae) trees as windbreaksin the cerrado. Florida Entomologist. 2015; 98(1):170-174.

60. Jansson J, Ekbom B. The effect of different plant nutrient regimes on the aphid *Macrosiphum euphorbiae* growing on petunia. Entomologia Experimentalis et Applicata. 2002; 104:109-116.

61. Nowak H, Komor E. How aphids decide what is good for them: experiments to test aphid feeding behaviour on *Tanacetum vulgare* (L.) using different nitrogen regimes. Oecologia. 2010; 163(1):973–984.

62. Han P, Lavoir AV, Le Bot J, Amiens-Desneux E, Desneux N. Nitrogen and water availability to tomato plants triggers bottom-up effects on the leafminer *Tuta absoluta.* Scientific Reports. 2014; 4(1):1-8.

63. Unsicker SB, Mody K. Influence of tree species and compass bearing on insect folivory of nine common tree species in the West African savanna. Journal of Tropical Ecology. 2005; 21(2):227-231.

64. Leite GLD, Veloso RV dos S, Martins ER, Zanuncio JC, Fernandes GW, Almeida CIM, et al. Population of herbivores insects on different sides of *Caryocar brasiliense* (Caryocaraceae) trees in the Brazilian Cerrado Region. Journal of Medicinal Plant Research. 2012; 6(43):5557-5565.

65. Zografou K, Adamidis GC, Komnenov M, Kati V, Sotirakopoulos P, Pitta E, et al. Diversity of spiders and orthopterans respond to intra seasonal and spatial environmental changes. Journal of Insect Conservation. 2017; 21(3):531-543.

66. Riihimäki J, Vehviläinen H, Kaitaniemi P, Koricheva J. Host tree architecture mediates the effect of predators on herbivore survival. Ecological Entomology. 2006; 31(1):227–235.

67. Auslander M, Nevo E, Inbar M. The effects of slope orientation on plant growth, developmental instability and susceptibility to herbivores. Journal of Arid Environments. 2003; 55(3):405–416.

68. Gagnon E, Brodeur J. Impact of plant architecture and extraguild prey density on intraguild predation in an agroecosystem. Entomologia Experimentalis et Applicata. 2014; 152(1):165–173.

69. Zalewski M, Hajdamowicz I, Stanska M, Dudek-Godeau D, Tykarski P, Sienkiewicz P, et al. β-diversity decreases with increasing trophic rank in plant – arthropod food chains on lake islands. Scientific Reports. 2018; 8(1):1-8.

70. Nascimento AR, Peronti ALBG, Kondo T. Two myrmecophilous scale insects, *Cryptostigma urichi* (Cockerell) (Hemiptera, Coccidae) and *Farinococcus multispinosus* Morrison (Hemiptera, Pseudococcidae), cohabiting inside branches of *Anadenanthera falcata* (Benth.) Speg. (Fabales, Fabaceae) in the Cerrado area of São Paulo State, Brazil. Revista Brasileira de Entomologia. 2012; 56(4):511-514.

71. Stewart-Jones A, Pope TW, Fitzgerald JD, Poppy GM. The effect of ant attendance on the success of rosy apple aphid populations, natural enemy abundance and apple damage in orchards. Agricultural and Forest Entomology. 2008; 10(1):37–43.

72. Zachariades C, Compton SG, Schatz B. Honeydew as danegeld? ants (Hymenoptera: Formicidae) tending a honeydew-producing Homopteran do not offer protection from its main natural enemies. Sociobiology. 2009; 54(2):471-488.

73. Kaminski LA, Freitas AVL, Oliveira PS. Interaction between Mutualisms: ant-tended butterflies exploit enemy-free space provided by ant-treehopper associations. The American naturalist. 2010; 176(3):321-334.

74. Karami-Jamour T, Mirmoayedi A, Zamani A, Khajehzadeh Y. The impact of ant attendance on protecting *Aphis gossypii* against two aphidophagous predators and it's roleon the Intraguild Predation between them. Journal of Insect Behavior. 2018; 31(2):222-239.

75. Tong H, Ao Y, Li Z, Wang Y, Jiang M. Invasion biology of the cotton mealybug, *Phenacoccus solenopsis* Tinsley: Current knowledge and future directions. Journal of Integrative Agriculture. 2019; 18(4):758-770.

76. Milton SJ, Dean WRJ, Plessis MA, Siegfried WR. A conceptual model of arid rangeland degradation. BioScience. 1994; 44(2):70-76.

77. Whisenant SG. Repairing damaged wildlands: A process-oriented, landscape-scale approach. Restoration Ecology. 1999; 9(2):249–249.

78. Alvares CA, Stape JL, Sentelhas PC, Gonçalves JLM, Sparovek G. Köppen's Climate Classification Map for Brazil. Meteorologische Zeitschrift. 2013; 22(6):711-728.

79. Santana PHL, Frazão LA, Santos LDT, Feranandes LA, Sampaio RA. Soil attributes and production of eucalyptus in monoculture and silvopastoral systems in the north of Minas Gerais, Brazil. Journal of Agricultural Science and Technology B. 2016; 6(1):361-370.

80. Kopittke PM, Menzies NW. A review of the use of the basic cation saturation ratio and the "ideal" Soil. Science Society of America. 2007; 71(2):259-265.

81. Krebs CJ. Bray-Curtis cluster analysis. 1. Biodiversity Pro Versão 2. 1989. Disponível em: <http://biodiversity-pro.software.informer.com/>; Acessado em: 02 de maio de 2019.

82. Hill MO. Diversity and evenness: aunifying notation and its consequences. Ecology. 1973; 54(2):427-432.

83. Jost L. Entropy and diversity.Oikos. 2006; 113(1):363–375.

84. Begon M, Townsend CR, Harper JL. Ecologia: de indivíduos a ecossistemas. 4. ed. Porto Alegre: Artmed, 2007. 752p.

85. Lazo JÁ, Valdés NV, Sampaio RA, Leite GLD. Diversidad zoológica asociada a um silvopastoreo leucaena-guinea con diferentes edades de establecimiento. Pesquisa Agropecuária Brasileira. 2007; 42(12):1667-1674.

86. Wilcoxon F. Individual comparisons by ranking methods. Biometrics Bulletin. 1945; 1(6):80-83.

87. SAEG - Sistema para Análises Estatísticas, Versão 9.1: Fundação Arthur Bernardes – UFV - Viçosa, 2007. Disponível em:<http://arquivo.ufv.br/saeg/>. Acessado em: 03 de Dezembro de 2017.

Table 1 – Numbers of leaves/branch, branches/plant, percentage of soil cover, abundance (Ab.), diversity index (D.) and species richness (SR) of phytophagous Hemiptera (Hem.), protocooper ants (Ants) and predators of Sternorrhyncha (Pred.Ster.) in *Acacia auriculiformis* (mean±SE) with and without application of dehydrated sewage sludge

	Dehydrated sewage sludge		Test of Wilcoxon	
	with	without	*VT**	*P*
Leaves/branch	35.00±1.03	28.23±0.89	4.2	0.00
Branches/plant	50.10±1.29	24.74±0.60	5.9	0.00
Percentage of soil cover	29.34±1.25	6.87±0.42	3.5	0.00
Ab. Hem.	8.17±1.96	3.29±1.46	3.3	0.00
D. Hem.	4.99±0.79	3.12±0.43	1.6	0.04
SR Hem.	2.38±0.27	1.34±0.20	2.8	0.00
Ab. Ants.	18.29±3.30	11.33±1.36	1.2	0.12
D. Ants	6.84±0.64	6.53±0.61	0.3	0.37
SR Ants	3.30±0.32	3.21±0.24	0.6	0.27
Ab. Pred.Ster.	1.54±0.29	0.83±0.18	1.8	0.03
D. Pred.Ster.	3.38±0.59	1.44±1.44	2.9	0.00
SR Pred.Ster.	1.08±0.17	0.59±0.10	2.1	0.02

n= 24 per treatment. VT*= test value

Table 2 - Relationships between abundance (Ab.) of protocooperating ants (Ants), predators of Sternorrhyncha (Pred.Ster.) and Phytophagous Hemiptera (Hem.); diversity (D.) of Pred.Ster.; species richness (SR) of Hem. and Pred.Ster. with leaves/branch (Nleaves) and/or branches/plant (Nbranches) and SR numbers of Hem. with RS of ants in plants of *Acacia auriculiformis*

Equations of principal component regression	ANOVA		
	R^2	*F*	*P*
Ab.Ants=-3.85+0.52xNleaves+0.40xAb.Hem.	0.16	4.12	0.02
SE Hem.=-0.70+0.38xSEAnts+0.04xNbranches	0.32	10.32	0.00
SE Pred.Ster.=0.18+0.02xNbranches	0.10	5.14	0.02
D. Pred.Ster.=-0.81+0.09xNbranches	0.60	23.59	0.00
Equations of simple regression analysis			
Ab. Pred.Ster.=0.01+0.001xNleaves	0.13	6.63	0.01
D. Pred.Ster.=28.22–1.85xNleaves+0.03xNleaves²	0.58	18.22	0.00
SE. Pred.Ster.=7.42–0.44xNleaves+0.08xNleaves²	0.13	3.57	0.04

ANOVA. n = 48, degrees of freedom: treatment = 1, repetitions = 22, and residue = 23.

Supplementary material I. Species of phytophagous Hemiptera, protocooperating ants, predators of Sternorrhyncha on *Acacia auriculiformis* plants

Phytophagous Hemiptera	
Achilidae	Non-identified
Aleyrodidae	Non-identified
Aethalionidae	*Aethalion reticulatum* L.
Cicadellidae	*Acrogonia* sp.
	Balclutha hebe Kirkaldy
	Erythrogoia sexguttata F.
Cicadidae	*Quesada gigas* Oliver
Coreidae	*Leptoglossus* sp.
Fulgoridae	Non-identified
Membracidae	Non-identified
	Membracis sp.
Nogodinidae	Non-identified
Pentatomidae	Non-identified
Scutelleridae	*Pachycoris torridus* Scopoli
**Predators of Sternorrhyncha**	
Coleoptera: Cantharidae	*Cantharis* sp.

Coccinellidae	*Cycloneda sanguinea* L.
Diptera: Syrphidae	*Syrphus* sp.
Dolichopodidae	Non-identified
Neuroptera: Chrysopidae	*Chrysoperla* sp.
Protocooperating ants	
Hymenoptera: Formicidae	*Brachmyrmex* sp.
	Camponotus sp.
	Cephalotes sp.
	Ectatomma sp.
	Pheidole sp.
	Pseudomyrmex termitarius Smith
